# Editorial: Non-communicable diseases; sexual reproductive issues; infectious diseases and health systems challenges

**DOI:** 10.4314/ahs.v22i4.1

**Published:** 2022-12

**Authors:** James K Tumwine

We are pleased to bring you this bumper December 2022 issue of *African Health Sciences*. It comes on the heels of the Climate change conference in Egypt and the HIV/AIDS day. Days that remind us that climate change is a real challenge and that the stemming of the HIV/AIDs pandemic is likely to slip through our hands unless there is an urgent reinvigoration of Africa's approach that was once the envy of the world in the last 20 years.

In this issue we bring you a salad of sorts! This includes papers on cancer^1^–^12^; diabetes mellitus^13^–^16^; surgery^15^–^25^; other non-communicable diseases^26^–^33^, as well as sexual reproductive health^34^–^46^. The menu continues with HIV /AIDS papers^47^–^54^. This is appropriate because 1^st^ December is World AIDS Day and countries in Sub Saharan Africa have little to celebrate as new infections and needless deaths continue in the face of global and local financial challenges. Clearly new strategies for engaging communities and governments to find resources badly needed for prevention and control are needed urgently. Development partners have an obligation to come to the support of low-income countries despite worldwide economic difficulties.

The infectious disease theme continues with papers^55^–^63^ on COVID-19 and other infections^64^–^69^. While we rightly put emphasis on infectious diseases, there is light at the end of the tunnel regarding the Ebola out break in Uganda: the country has spent about 3 weeks without any new cases of Ebola. In fact, the Ministry of Health has recently alluded to the fact that there are zero case of Ebola in the country: good news! We end the treatise with papers on childhood illnesses^70^–^74^ and health systems^75^–^77^.

Please relax and enjoy this Christmas issue of African Health Sciences as you reflect on the issues that give us sleepless nights in Africa and the world: conflict, preventable diseases; climate change, and concomitant famine. If we made individual and collective efforts, together we would succeed in making this fragile planet a safe and enjoyable place to live.

Merry Christmas!
